# Miscellanies

**Published:** 1837-04-01

**Authors:** 


### MISCELLANIES.

### Medical Attendance on the Sick Poor.

I. Observations on the Arrangements connected with the Relief of the Sick Poor; in a Letter to the Right Honourable the Lord John Russell, &c. &c. By J. Yelloly, M.D. &c. &c. 8vo. Stitched, pp. 43. Longman's, 1837.

II. Observations on the Present Condition of Medical Relief for the Sick Paupers; with Recommendations yor an Altered and Improved System.

In a former Number of this Journal,* we took up the subject of the medical section of the New Poor Law, and recommended our professional brethren to bestir themselves, and by urgent and judicious appeals to the legislature to procure better treatment at its hands. Our feelings on this subject have certainly not diminished, but acquired force from reflection and time. The mass of practitioners are deeply sensible both of the injury and the insult heaped upon them, and, throughout the country, they are protesting against the arbitrary, we may say the insolent conduct of the Poor Law Commissioners.

The Weekly Medical Journals are incessantly occupied with the discussion of the Poor Law, and with loud denunciations of its authors and its principles. Not confining themselves to the regulations affecting the profession, they step out of their sphere, we think most injudiciously, and mix themselves up with opposition to the principle of the Poor Law Bill.

The pamphlet before us, from the pen of Dr. Yelloly, applies itself to the subject of medical relief to the sick poor, and carefully avoids the general question. Dr. Yelloly himself, appears in an amiable light, for his interests are not and cannot be involved, and zeal only for the wider interests of his brethren can tempt him to the arena of political dispute.

From this pamphlet we shall make some extracts, descriptive of the evils, and suggesting the remedy, of the existing system: and we shall add the propositions which have emanated from some of the most indefatigable reformers of its abuses.

### *Applicability of the Principle of Competition?*

If a man wishes a building erected, he puts the thing up to competition, and decides by tender. But he does not necessarily take the lowest tender, indeed the lowest tender is notoriously usually rejected. A very low tender implies fraud, and the suspicion of that occasions the rejection. But whatever tender is accepted, a guarantee is obtained for the bonâ fide fulfilment of the stipulations, for an architect or surveyor is appointed to see them properly executed.

This principle of competition, this mode of purchasing cart loads of pork or of brick and mortar has been liberally applied to the acquisition of medical attendance on the poor. The Commissioners have demanded tenders, have selected low ones, and have entrusted the sick pauper to the contractor they have chosen. But where is the guarantee for the performance of the contract? Where is the professional surveyor who shall pronounce that due medical attention, proper drugs, or skilful service have been given? The Commissioners have no such surveyor in their scheme. To compensate for that, they have a method of their own. A relieving officer, no medical man, and a Board of Guardians, not medical neither, decide both the necessity for medical attendance, and its propriety when given. An admirable and a humane provision, distinguished alike for its sagacity and feeling. A provision which, in point of common sense, is about on a par with putting a butterman, to decide on the execution of the contract of a bricklayer.

* No. 50.

"No one," observes Dr. Yelloly, "would, I am sure, make competition and low charges, ingredients in his choice of a solicitor, who is to be entrusted with the management of his most important private affairs; or of an engineer, who is to devise or to carry into effect momentous operations, whether of a public or private nature; or of the fabricator of that nice and curious machinery, which, in the trackless ocean, enables man to pursue his way with confidence and accuracy; but his aim would be, by a judicious selection in the first place, and liberal treatment in the next, to ensure the full exercise of the most faithful, and most able services.

In all these cases (and it is hardly necessary to multiply them, or to deduce examples from government, war, or diplomacy) it is not a common operation which is required, or an article of obvious character which is to be provided; but it is the intellect, the spark of divine essence which we wish to enlist in our service; the exercise of unfettered zeal, assiduity, and talent, which would be chilled and repressed by anything like illiberality and suspicion."

"And yet with regard to the parish surgeon, he is required either to accept a sum which the least consideration or inquiry will evince to be totally inadequate; or he is desired to give in his proposals, which he knows must be within certain prescribed limits; or he is threatened with a competition, by the introduction of some new practitioner into his district, which must in some degree risk a still further reduction of his hardly earned income, than the crowded state of the profession has yet occasioned. There is no disposition to reward according to services, but the ungenerous feeling is too generally entertained, to employ the very hardships under which the profession labours by the numbers who enter it, as a means of still further depression. I have remarked that the parsimony is inconsistent, and that it applies to an article of comparatively small amount; and I will add, that if medical attendance were even adequately remunerated, it would still form but a small item of general parochial expenditure."

Would these "tender" Commissioners adopt this plan, were their own well-lined paunches the seat of colic or of flatulence? Not they. No "cheap and nasty" surgeons or physic would enter either their doors or stomachs, but disregarding the principle of competition and of tender, they would consult whom they thought the ablest, not the neediest man. The same thing would be done, and is done constantly, throughout society. It is not *mere* cheapness that the anxious mother, husband, or child consults in procuring medical assistance for the objects nearest and dearest to their hearts. The pauper, however, is treated otherwise. He must have medical relief. That is a bore. Let him have the worst.

It would be absurd to demand on the part of the Commissioners any sympathy with the profession itself. They cannot be expected to feel any regard for its interests or its respectability. When they are told then, that to be a parish surgeon is now almost discreditable; when they learn that taking office is a bitter pill, swallowed only through dread of a worse, they naturally reply, "What is that to us?" They are in their seats for the purpose of economy; their great aim is to lower the rates. If a pauper or two die from tardy or from incompetent medical assistance, or if a liberal profession is bullied and degraded, those are small matters in comparison with the solid savings on the poor's books. Yet it must be owned that the profession *is* degraded, and that the appointment of a parish surgeon has lost, not gained, in respectability. Dr. Yelloly shall again speak, and from personal experience.

"I may add, that the inadequate terms of remuneration, give a low rank to parish employment, and prevent the more established and best estimated practitioners of a district, from wishing to have any share in it.

Of this circumstance I know personally several examples; and cannot help feeling it to be a subject of great regret, that any want of liberality in medical arrangements, should be the means of depriving the poor of the best advice which their locality can afford them."

"In Norwich, they have had continual changes. At first they had the attendance of physicians; but no one of that class will now accept office under this system; and they are entirely dependent on the younger class of practitioners, who may be expected to throw up their appointments after a certain time; and this when the addition of one hundreth part (£200) to the annual expenditure, calculating it at an average of £20,000. per annum, would render the attendance on the poor very satisfactory."

"In my own neighbourhood, in the small town of Beccles and Bungay, the principal practitioners do not take parochial employment; and this is likewise the case at Norwich."

Whatever the Commissioners may say or think, their employers, the Government, will find, that to goad an entire profession into open hostility against a law, itself not popular, is any thing but politic—and that the attempt at once to insult, and by the operation of terror, to silence that profession, will most assuredly fail. Either the terms offered it are just or not. If they were just, the feeling that exists could never have been raised—and if they are unjust, neither authority nor cajolery can stifle it.

The profession loudly complains that it is snubbed by every Board of Guardians, nay, by the very relieving officer—that Government encourages a ruinous and derogatory competition—and that the attempt to procure fair and reasonable terms is put down, by the threat of procuring young adventurers, the—

Heaven's Swiss, who fight for any God or man—

to bully the respectable practitioners.

It is too much for our patience when the Commissioners reply—that they entertain no intention of insulting the profession—that medical men are the best judges of what they can or cannot afford, and that acceptance of terms must be construed as approval of them—and that the *honour* of the office of a parish surgeon satisfactorily accounts for the readiness to take it at a loss. The fallacy of these arguments, and the solemn sarcasm they carry, would be ridiculous, if the subject were not too serious for a joke.

But what is to be done?

Dr. Yelloly proposes:—

1. That the parish surgeon should be elevated, not degraded—his utility enhanced by self-respect, not diminished by contumely and wrong. Such an argument is probably unintelligible to the Commissioners.

"With regard to the mode in which a change in the medical arrangements could be effected, it would not be difficult to assimilate the system of providing medicines for parishes, in some degree with the method pursued in furnishing them for the army and navy, or for hospitals and dispensaries. It would be important to banish entirely the present plan of making it the interest of a professional man to withhold the medical aid that is proper; and this it appears to me, may be readily effected, either by providing parishes or unions, with a certain supply of medicines, and awarding a remuneration for attendance separately; or if it be determined that one sum shall still cover the whole, by making a proper estimate of the value of the medicines necessary, and superadding a fair remuneration for attendance and trouble.

Such an arrangement would be most readily effected with the sanction of Government, through the means of the Poor Law Commissioners. But it is a matter of grave and important consideration, whether it would not be in proper conformity with other parts of our social system, that there should be a medical gentleman of character and experience, connected with the Poor Law Commission, and possessing the confidence of the public and the profession, to study and advise on the many subjects of a professional nature which must continually come before that Board. While parishes acted separately, such a plan was impracticable; but now that the management of the poor is concentered in one body, I do not think the medical department can be efficiently, or satisfactorily regulated, without such aid. The Commander-in-Chief of the Army, or the Lords of the Admiralty, would never think of trusting solely to their own knowledge of medical affairs, for managing concerns which relate to the health of our soldiers and sailors. They depute this duty to adequate and efficient men, who, while they leave the whole power of the general regulating body untouched, are the confidential advisers of the heads of departments, and are a guarantee to the public and the profession, for their having every species of professional information placed before them to direct their decisions."

We leave Dr. Yelloly, and turn to a letter published by the Messrs. Rumsey, and Mr. Robert Ceely. It breathes a tone of indignant remonstrance—that is natural; points out the evils of the present system, and the abused powers of the Commissioners—that is now unnecessary; and proposes remedies, which we shall quote.

1. "So long as the present unlimited powers of appointing and dismissing—of summoning and judging—of justifying and condemning medical practitioners, continue to be vested in parties necessarily so incompetent, (even although a fairer remuneration for the duties performed were conceded) the medical department of the Poor-law must continue a fruitful source of degradation to the profession—of contention and complaint in every locality—and of suspicion and dissatisfaction, not only towards the Poor-law administration, but towards the government of the country.

It has long been found expedient that medical men employed in the public service should be placed under medical supervision. For this reason, and to remedy the serious evils just noticed, as well as to introduce some uniformity and regularity into the medical arrangements for the sick poor, we recommend that the general superintendence and control of the medical department of the Poor-law administration be entrusted to a medical board, similar to the army and navy medical boards, and composed of persons practically acquainted with medico-parochial duties."

2. "We propose either that the legislature, as a supreme impartial authority, should fix the rate of remuneration; or that this be entrusted to a Board, to whom both parties might confidently look for justice.

If the latter alternative were adopted, and if it were agreed that the Medical Board, already recommended for other purposes, should also exercise this important function, it would be reasonably expected by the medical profession, one member at least of this Board should be appointed by, and responsible to the profession."

3. "At present, weekly medical returns are required to be made to the Board of Guardians, with specifications, not merely as to the names, ages, date of illness, and general state of the pauper patients, which is an unobjectionable regulation, but also as to the nature and designation of the illness, the frequency of medical visits, and even the treatment of the disorder, which latter three particulars must be, for the most part unintelligible to the parties by whom they are required, and are felt, on this ground, to be inquisitorial by the medical attendants.

While, therefore, the first four particulars continue to be reported to the Boards of Guardians, it is highly important that complete reports of cases under treatment be made to the proposed Medical Board. Such reports would afford evidence of the nature and amount of duty, and the efficiency of its performance; they would also constitute a check against abuses of every description; and if periodically completed and published by the Medical Board, would prove valuable to science, and useful to the public."

4. "Under the present system it is a common practice to entrust the care of extensive districts of parishes, and sometimes of entire unions, to individual medical officers. By this arrangement, so detrimental to the sick poor, and so injurious to established practitioners, the Poor-law authorities are enabled to offer "wider fields" for competition, and thus to attract a greater number of unemployed adventurers, and to reduce still further the rate of remuneration. But however destructive to the interests of the present race of practitioners, and to the safety of sick paupers, such temporary expedients may prove, it is obvious that, ultimately, there can be no other reasonable method of supplying medical aid to the paupers, but by means of the nearest medical residents—men whose ordinary range of practice includes the parish for which a medical officer is required.

We therefore consider it essential that the present arbitrary distribution of the parishes into medical districts be abolished, so that the medical officer be appointed for each district separately.

No surgeon should be permitted to undertake more than a certain amount of parochial duty, nor to act unless possessing certain qualifications as to length of practice, &c. &c.; both which should be regulated by the Medical Board.

If any surgeon, not previously resident or living at a distance, be appointed to the care of a parish or workhouse, the reasons for such appointment should be transmitted to the Medical Board. No election to be valid unless confirmed by the Medical Board. No medical officer should be liable to any investigation of his official conduct, or to be dismissed from office, except by the Medical Board.

Every medical officer should be permitted to resign at any time, upon giving reasonable notice."

5. The Relieving officer is obviously unable to decide on cases fit or unfit for medical relief.

"We propose, therefore, that the present practice be entirely discontinued, as affording no security to the rate-payers, no safety to the sick paupers, and no justice to the medical officer, and that the following arrangement be substituted :—

Every sick pauper to apply in the first instance to the medical officer, who should be authorized to relieve him at once, if necessary, and, in that case, to furnish him with a certificate stating that the case required treatment. This certificate should be sent by the patient to the relieving officer in a specified time after receiving it—say twenty-four hours. The relieving officer should enquire into the circumstances of the patient (if he be not already receiving relief in money or in kind) before the next meeting of the Board of Guardians, and should make a report thereon at that meeting; and thus enable the Board to divide the patients attended into two classes—the one to which the medical relief should be unconditionally given, and the other to which it should be merely afforded by way of loan, the cost being recoverable according to the provisions of the Poor-Law Amendment Act. The Board of Guardians might, of course, if they thought proper, refuse the continuance of the loan of medical relief to such persons. The cost of the relief thus afforded might be calculated at so much *per diem*, with an additional charge for journeys; and should be distinct from any arrangement for the regular paupers."

At present a Committee of the House of Commons is sitting on this subject. Before that Committee the question will be decided, not by passion, but by reasoning and facts.

Now is the time for actions instead of words! *Medical* evidence has never been considered as the very best in the jury-box, and sincerely do we hope that the witnesses called before the parliamentary committee will store their minds with specific instances that have occurred under their own eyes, and not trust to hear-say evidence which will go for nothing at all—or worse than nothing, because it will prejudice the cause in which they are engaged. The witnesses must recollect that at least four-fifths of the committee are favourable to the principle of the Act, and, to a certain extent prejudiced in favour of its details. Their evidence will therefore be sifted very minutely—and more especially the evidence of those whose positions bring their pecuniary interests in collision with the actual working of the new system. It would, indeed, be of the utmost importance if a number of medical practitioners, quite beyond the pale of suspicion as to private interest, but who have observed the evils of the New Act, were to come forward on the present occasion, to state the results of their observations.

We would say to our professional brethren, be determined, but be cautious. Do not look merely to your own narrow interests, but invoke the eternal principles of justice and humanity.

One word before we quit the subject. Our contemporaries have acted most unwisely in mixing up the general and medical question. They may feel assured that most men of education, of reflection, and of respectability are agreed in the reprobation of the principle of the Old Poor Law, and in the approval of that of the New. The great opposition to the latter has arisen from discontented jobbers, or from persons whose good feelings exceed their good sense. The violent denunciations of our contemporaries have helped to place the profession in both categories.

---

### Poison of Pork.*

At various times the poison of fish, of sausages, and of pork, has attracted the attention of the profession. Some of our German contemporaries have imagined that the poisonous effects of sausages resulted from the development of a peculiar fatty acid—while those of cheese depended on excess of caseic, and the presence of sebaic acid. But, as the above and other substances prove poisonous to some people, and are innocuous to others, Dr. M'Divett is inclined to suppose that, " in certain states of the digestive organs the articles of food, or some portion of their elements, are converted into poisons by admixture with morbid secretions from the stomach or intestinal canal, or simply by some derangement in the process of digestion." This is an opinion which, of course, admits not, at present, of direct proof. We know that fresh pork is apt to disagree with some people, producing diarrhœa, griping, or vomiting—imitating the action of some of the more violent poisons. Dr. M'Divett alludes to the ludicrous case related by the late Gregory of supposed gout in the stomach, which turned out to be a large mess of pork dislodged by an emetic. But we shall now notice one or two of the examples brought forward by Dr. M'D.

*Case* 1. A person conceived he had been poisoned by a young woman, whom he accused of this attempt. The belly was swollen—acute pain in the epigastrium—constant vomiting of dark-coloured fluids—burning sensation in the throat—pulse quick and faultering—cold extremities, &c. An emetic was administered, which brought off some lumps of half-digested meat, with immediate relief of the symptoms, and dispersion of the poison. It turned out to be pork that this person had eaten rather too plentifully.

*Case* 2. A plethoric but hale man was attacked with crampy pains in the stomach, at three in the afternoon, which became of a more violent kind. The skin was covered with cold sweats, the pulse weak and fluttering. He had dined on *pork-fry*. An emetic quickly relieved him, and brought up several pieces of fat pork and pig's-liver.

*Case* 3. Dr. M'D. was called to a middle aged man who had been suddenly attacked with excruciating pain in the region of the duodenum, followed by vomiting of acrid matters. He was apparently in a very dangerous state, with symptoms resembling those of a perforated intestine. It was ascertained that he had eaten roast pork on the preceding day. Half a drachm of ipecacuan produced copious vomiting, by which bile and some bits of half-digested pork were brought up. The medicine operated downwards also, and complete relief was obtained. The family had partaken of the same food, without inconvenience.

*Case* 4. In January, 1836, a man had dined on pork at two o'clock, and a few hours afterwards was seized with agonizing pain in the ileum, vomiting, &c. On the following morning the pain continued, and the vomiting was urgent, with swollen and tender abdomen, and an urticarious rash over the breast, legs, and arms. An emetic was exhibited, which operated upwards and downwards, with immediate relief.

Two or three other cases are detailed by Dr. M'Divett, followed by some very sensible observations, which are well deserving the attention of the profession.

---

* Dr. M'Divett. Ed. Journal, October, 1836.

